# A developmental approach to diversifying neuroscience through effective mentorship practices: perspectives on cross-identity mentorship and a critical call to action

**DOI:** 10.3389/fnint.2023.1052418

**Published:** 2023-02-08

**Authors:** Tanisha G. Hill-Jarrett, Rowena Ng, Carlos Cardenas-Iniguez, Jemima Akinsanya, Ismary Blanco, Johnathan M. Borland, James S. Brown, Tameka Clemons, Adriana K. Cushnie, Jacqueline Garcia, Brianna George, Cera W. Hassinan, Timothy J. Hines, Dan Landayan, Taylor A. McCorkle, Katherine R. Meckel, Mariajose Metcalfe, Samantha A. Montoya, Deborah K. Rose, Desmond R. Warren

**Affiliations:** ^1^Memory and Aging Center, Department of Neurology, University of California, San Francisco, San Francisco, CA, United States; ^2^Department of Neuropsychology, Kennedy Krieger Institute, Baltimore, MD, United States; ^3^Department of Psychiatry and Behavioral Sciences, Johns Hopkins University School of Medicine, Baltimore, MD, United States; ^4^Department of Population and Public Health Sciences, Keck School of Medicine, University of Southern California, Los Angeles, CA, United States; ^5^Neuroimmunology Clinic, National Institute of Neurological Disorders and Stroke, Bethesda, MD, United States; ^6^Interdisciplinary Program in Neuroscience, Georgetown University, Washington, DC, United States; ^7^Department of Neuroscience, University of Minnesota, Minneapolis, MN, United States; ^8^Department of Psychology and Program in Neuroscience, Florida State University, Tallahassee, FL, United States; ^9^Department of Professional and Medical Education, Meharry Medical College, Nashville, TN, United States; ^10^Department of Neuroscience, Tufts University School of Medicine, Boston, MA, United States; ^11^Department of Physiology and Pharmacology, School of Medicine, Wake Forest University, Winston-Salem, NC, United States; ^12^Basic Sciences Division, Fred Hutchinson Cancer Research Center, Seattle, WA, United States; ^13^Molecular and Cellular Biology Program, University of Washington, Seattle, WA, United States; ^14^Jackson Laboratory, Bar Harbor, ME, United States; ^15^Department of Psychiatry and Behavioral Sciences, Stanford University, Stanford, CA, United States; ^16^Department of Neurobiology and Anatomy, Program in Neuroscience, Drexel University College of Medicine, Philadelphia, PA, United States; ^17^Department of Biology, Swarthmore College, Swarthmore, PA, United States; ^18^The Friedman Brain Institute, Department of Neuroscience, Icahn School of Medicine at Mount Sinai, New York, NY, United States; ^19^Department of Anatomy and Neurobiology, University of California, Irvine, Irvine, CA, United States; ^20^Department of Psychiatry and Behavioral Sciences and Department of Psychology, Graduate Program in Neuroscience, University of Minnesota, Minneapolis, MN, United States; ^21^Department of Neurology, Duke University School of Medicine, Durham, NC, United States; ^22^Department of Psychology, Georgia State University, Atlanta, GA, United States

**Keywords:** perspective, mentorship and early career scientist challenges, neuroscience, diversity in science, cross identities, academic pipeline, qualitative survey

## Abstract

Many early-career neuroscientists with diverse identities may not have mentors who are more advanced in the neuroscience pipeline and have a congruent identity due to historic biases, laws, and policies impacting access to education. Cross-identity mentoring relationships pose challenges and power imbalances that impact the retention of diverse early career neuroscientists, but also hold the potential for a mutually enriching and collaborative relationship that fosters the mentee’s success. Additionally, the barriers faced by diverse mentees and their mentorship needs may evolve with career progression and require developmental considerations. This article provides perspectives on factors that impact cross-identity mentorship from individuals participating in Diversifying the Community of Neuroscience (CNS)—a longitudinal, National Institute of Neurological Disorders and Stroke (NINDS) R25 neuroscience mentorship program developed to increase diversity in the neurosciences. Participants in Diversifying CNS were comprised of 14 graduate students, postdoctoral fellows, and early career faculty who completed an online qualitative survey on cross-identity mentorship practices that impact their experience in neuroscience fields. Qualitative survey data were analyzed using inductive thematic analysis and resulted in four themes across career levels: (1) approach to mentorship and interpersonal dynamics, (2) allyship and management of power imbalance, (3) academic sponsorship, and (4) institutional barriers impacting navigation of academia. These themes, along with identified mentorship needs by developmental stage, provide insights mentors can use to better support the success of their mentees with diverse intersectional identities. As highlighted in our discussion, a mentor’s awareness of systemic barriers along with active allyship are foundational for their role.

## Introduction

The lack of diverse representation in neuroscience remains a significant problem reflecting the systemic inequities of the United States (U.S.) educational system and structural oppression that is deeply entrenched into the fabric of the country. Current data from the National Center for Education Statistics between the years of 1995–2015 show that non-Latinx, white neuroscience graduates represent the largest percentage of graduates across bachelor’s (52.6–66.3%), Master’s (52.6–60.8%), and doctoral (53.2%-66.5%) degree granting neuroscience programs (Ramos et al., 2017). While the number of neuroscientists entering the field is growing, the proportion of those from underrepresented backgrounds remains markedly lower than the U.S. census representation.

Historically, research efforts to characterize the demography of the academic landscape have focused on very narrow aspects of diversity (e.g., race, *or* gender, *or* disability). However, there is growing acknowledgment of the expansiveness of identity and its intersections. These intersecting identities shape access to power/privilege and, resultantly, one’s experience navigating the world, including academia (Crenshaw, 1989; Cole, 2009). Transgender and nonbinary/gender expansive people, undocumented immigrants, people living with a disability, and religious minorities have been systematically excluded in the neurosciences, and initiatives to improve their representation in academic spaces are lacking (Corrington et al., 2020). The inclusion of diverse voices in the neurosciences is part of the necessary shift towards a more equitable society and directly contends with the discrimination and oppressive hegemony entrenched within the institution of education (Zhao, 2016; Bartz and Kritsonis, 2019). Additionally, diverse perspectives are the catalyst for innovation in the sciences (Graham et al., 2020; Daehn and Croxson, 2021) and contribute to more dynamic problem solving (Friedman et al., 2016).

Diverse identities should be reflected within all levels of the neuroscience pipeline, but representation appears poorest at higher ranking positions—that is, the further you advance along the academic pipeline, the “leakier” it becomes (Shaw et al., 2021). The attrition, or leaks, of diverse neuroscience talent tends to occur at critical transition junctures involving academic advancement (Shaw et al., 2021). A number of factors—likely interacting with one another—impact retention of diverse talent and contribute to leaks. While a comprehensive overview of factors contributing to attrition is beyond the scope of this article, we offer a few examples across different stages of training and career.

Among first-generation college students, some face unique challenges including disparities in access to resources and financial precarity impacting their ability to complete a degree. First generation and low income students are less likely to complete an application for financial aid (Bahr et al., 2018) and there is evidence that financial aid offer letters use inconsistent terminology and conflate different aid options (Burd et al., 2018), creating barriers to appropriate decision making to fund college education. Current data also show bias in graduate school admission processes including the discriminatory use of the standardized testing like the Graduate Record Exam (GRE) or other quantitative measures (e.g., GPA) as a predictor of academic success despite the GRE having low predictive validity (Moneta-Koehler et al., 2017). The GRE is a poor indicator of future research productivity (Woo et al., 2022) or graduate degree completion (Petersen et al., 2018) as it instead measures test taking ability and exam familiarity (Kruse, 2016). The continued use of the GRE by admissions committees contributes to pipeline leakiness as its incorrect use as an indicator of aptitude most often unfairly excludes women, racially and ethnically minoritized persons, and those from socioeconomic disadvantaged backgrounds from admission into graduate school programs (Miller and Stassun, 2014).

For historically excluded and marginalized graduate students, socialization to academia (i.e., the process whereby institutional values, skill sets and ways of engagement are learned and reinforced) can be in direct conflict with existing belief systems and often upholds dominant cultural norms (Weidman et al., 2001; Azizova, 2016), impacting feelings of belongingness and inclusivity both of which are predictors of retention and success in STEM PhD programs (Fisher et al., 2019). For women and birthing people, a critical transitional period along the academic pipeline postdoctoral phase where roles and responsibilities as a parent and scientist may collide with very little leeway and support. In fact, the decision of whether to start a family and when is a major predictor of attrition among postdoctoral fellows (Resmini, 2016; Ledford, 2017; Ysseldyk et al., 2019).

At the faculty level, funding rates are significantly lower for racially and ethnically minoritized groups and data show the Black scientists are less likely than their peers to receive an R01 grant from the NIH (National Institute of Health), reflective of a substantial funding gap impacting productivity and career progression on the tenure track (Ginther et al., 2011; Wilson et al., 2018). One study reported that applications from Black scientists were less likely to be discussed and received lower impact scores (Hoppe et al., 2019). In the study, greater than 20% of the funding gap was attributable to differences in choice of research topics by Black scientists compared to white counterparts. Topics more commonly identified as relevant to Black scientists, such as community-engaged research and population health, were awarded at lower rates, demonstrating bias and discrimination in funding priority as well as epistemic exclusion devaluing and delegitimizing the important work of racially minoritized scholars (Settles et al., 2021). The unfortunate truth of these data is that the Ivory Tower remains unattainable, unwelcoming, or housed with a glass ceiling that limits the upward mobility of many diverse neuroscientists [e.g., see Black in the Ivory (Davis, 2021) for overview].

While diversity is slowly increasing at the student level, those who hold high ranking faculty positions do not reflect the same diverse demographic and social identities. Often, cisgender, heterosexual, white men occupy senior neuroscience positions and serve as gatekeepers to academic advancement thereby limiting upward mobility. Not only does a power differential exist within the mentorship relationship based on career status, but the mentor’s status within society’s social hierarchy can influence the mentorship dynamic (Ragins, 1997; Thorne et al., 2021). As diverse neuroscience mentees ascend the academic ranks, it is likely that they will encounter incongruence between their identity and the social-demographic characteristics of a mentor. Admittedly, a number of thriving cross-identity mentoring relationships exist. In some instances, however, it may affect the mentor’s ability to effectively respond to the systemic oppression, discrimination, and other challenges uniquely faced by the mentee.

Lack of effective mentorship has been regularly cited as a barrier to successful advancement in neuroscience (Singleton et al., 2021), and academia more broadly (Davis et al., 2021; Ocobock et al., 2021). Thus, it is incumbent on mentors to maintain awareness of approaches that are less helpful in mentorship dyads where identity incongruence is a factor, and to apply strategies and mentorship styles that facilitate retention and progression within neuroscience. For instance, in a study examining cross-racial mentoring of racially and ethnically minoritized faculty, the mentor’s awareness of the mentee’s cultural experience, the mentor’s open-mindedness, and trust and comfort in the relationship shaped how race affected the relationship (Thorne et al., 2021). To date, there is limited research on mentorship practices that support diverse neuroscientists across training and career level. Identifying strategies that promote mentee retention and academic enrichment *across developmental stages* of education and career is central to building supportive, customized experiences unique to the mentee’s needs at each transition juncture. Accordingly, our manuscript seeks to expand extant work by applying a developmental framework to examining cross-identity mentorship factors that: (1) hinder, (2) support, and (3) retain diverse neuroscientists in academia across varied developmental stages of training and career.

## Methods

All study procedures were approved by the University of Minnesota Institutional Review Board and all participants provided written informed consent. Participants completed the survey on a voluntary basis and were offered co-authorship for their contribution.

### Participants

Study participants are members of the first cohort of Diversifying the Community of Neuroscientists (Diversifying CNS) program (Diversifying the Community of Neuroscientists, 2022)—a National Institute of Neurological Disorders and Stroke (NINDS) R25 funded initiative. Demographic data are presented in Table 1. Out of the 19 program participants, 14 (73%) responded to the survey online. Of this subgroup, the majority of respondents were graduate students (8/14; 57%), followed by early career faculty (4/14; 29%), and postdoctoral fellows (2/14; 14%). All respondents were affiliated with an academic medical institution or university and identified as first-generation graduate students.

**Table 1:** Demographic characteristics of Diversifying CNS survey respondents (*N* = 14).

	***n***	**%**
**Race**		
White	3	21.4%
Black	6	42.9%
Asian	1	7.1%
American Indian or Alaska Native	1	7.1%
Multiracial	3	21.4%
**Ethnicity**		
Hispanic/Latino	4	28.6%
**Gender identity**		
Cisgender female	10	71.4%
Cisgender male	4	28.6%
**Sexual orientation**		
Heterosexual	9	64.3%
Bisexual	2	14.3%
Queer	1	7.1%
Panromantic, gray asexual	1	7.1%
No response	1	7.1%
**Place of birth**		
United States	10	71.4%
Latin America	2	14.3%
Jamaica	1	7.1%
Germany	1	7.1%
**Professional background**		
Molecular/cellular neuroscience	10	71.4%
Behavioral neuroscience	2	14.3%
Neuropsychology	2	14.3%
**First generation American**	3	21.4%
**Person with a disability**	3	21.4%
**From economically disadvantaged background**	7	50.0%
**Primary language**		
English	12	85.7%
Spanish	2	14.3%

### Survey procedure

The online survey was designed by coauthors (TH-J, RN) who participate in the Diversifying CNS Program. Survey questions were developed based on review of literature on mentorship and diversity in academia and the workforce. Based on the literature, TH-J and RJ first identified themes and concepts that were important to capture with the survey questions. Next, a list of potential questions were generated and piloted with a person outside of the Diversifying CNS program to ensure readability and adequate functionality of the online survey platform. The final survey consisted of 27 open-ended questions and 17 quantitative questions distributed for online completion using Qualtrics. Participants were asked to respond about their experiences at their current stage of training or career. Examples of open-ended questions included:

What are your specific mentorship needs at this stage of your career?

Have there been any barriers in identifying a mentor who shares some aspect of your social or demographic identity?

What are the benefits to having a mentor whose identity differs from yours?

Additionally, participants provided written examples of both a good and bad mentorship experience, barriers within the relationship, and suggestions for improvement. Lastly, respondents were provided with a menu of options of mentorship features they may seek (e.g., role model for work/life balance, sponsorship, one-on-one time, source of support) and ranked the list in order of importance.

### Analysis

Narrative responses from the online survey were uploaded and organized in ATLAS.ti (Mac Version 22.1.0; ATLAS.ti, 2022). Responses were analyzed using an inductive thematic analysis (Braun and Clarke, 2006). This method is inductive, or data-driven, in nature and produces codes that directly reflect the data and are free of pre-existing theory. We were interested in understanding the lived experiences and meaning making of the group within a broader social and societal context and therefore prioritized the meanings derived from respondents’ words. TH-J and RN separately read and completed open coding of responses. Codes were independently assigned by the level of meaning—that is, text segmentation was completed by meaning conveyed and not line, sentence, or paragraph—so long as the essence of the idea was preserved (DeCuir-Gunby et al., 2011). Coders met to discuss their codes and generate a code book with formal code definitions and coding criteria. They reviewed the text and reassigned codes according to these criteria. Text was allowed to have more than one code assigned. TH-J and RN then identified codes that had conceptual similarity; these were grouped into a theme with subordinate subthemes.

Quantitative and descriptive data were analyzed using IBM SPSS 28.0 (IBM Corp, 2021). Between-group comparisons of continuous data were made across education/career level using ANOVAs. Statistical significance was set at *p* < 0.05. All survey respondents were provided with a draft of the manuscript, and revised according to their suggestions, to ensure their words were accurately reflected and anonymity was upheld.

## Results

### Quantitative

Respondents were in their current academic role for approximately 3 (*M* = 3.08, *SD* = 1.21) years, and the amount of time did not differ by career level, *F*_(2,11)_ = 2.27, *p* = 0.150. Early career faculty reported having significantly less frequent beneficial mentoring relationships in their current academic stage (44.5% of relationships are beneficial) compared to postdoctoral fellows (90.0%) and graduate students (80.0%), *F*_(2,11)_ = 9.22, *p* = 0.004.

### Qualitative

#### Factors impacting experience in neuroscience

**Figure 1:**
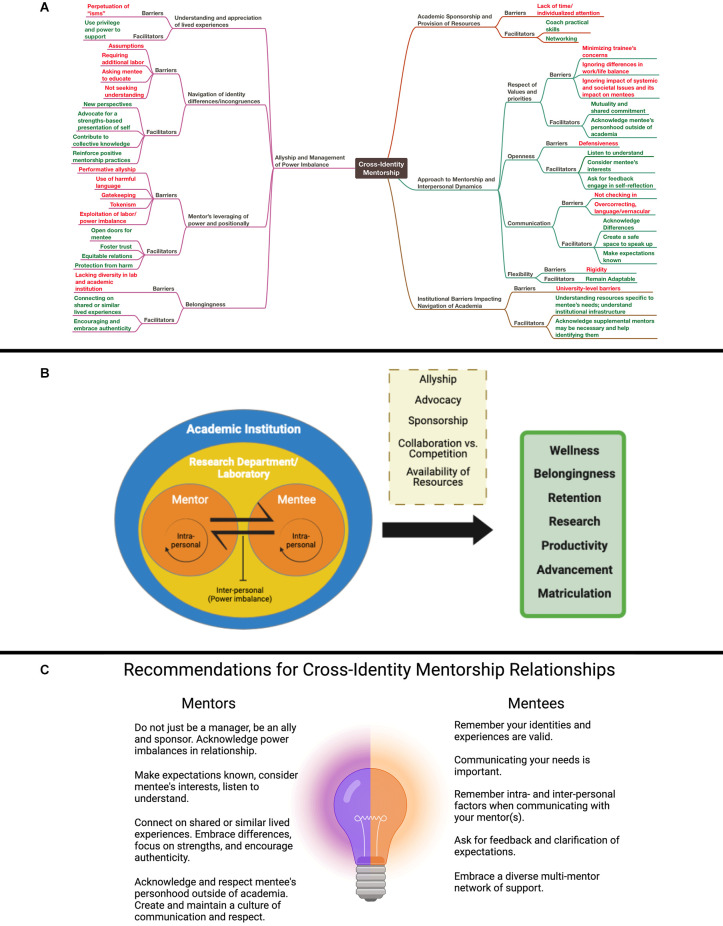
Factors related to cross-identity mentorship in the neurosciences. **(A)** A thematic map of barriers and facilitators in cross-identity mentor-mentee relationships. A full description with participant quotes is included in the Supplementary Table. **(B)** Factors impacting cross-identity mentor-mentee relationships depend on a number of layered structural, local, intra- and interpersonal factors, but are all important for the wellbeing, productivity, and success of mentees. **(C)** Recommendations for mentors and mentees that emerged from the early career neuroscientist survey.

Analysis of the narrative responses across the entire group revealed four themes that impact a mentee’s experience in neuroscience regardless of developmental stage: (1) approach to mentorship and interpersonal dynamics, (2) allyship and management of power imbalance, (3) academic sponsorship, and (4) institutional barriers impacting navigation of academia. The Supplementary Table summarizes themes and subthemes, and offers illustrative quotes of barriers and supportive practices. Figure 1A provides a thematic map of the qualitative results.

##### Theme 1: approach to mentorship and interpersonal dynamics

For a majority of respondents, the mentor’s approach to engaging was important and impacted their perception of the relationship.

Respect of values and priorities: Respondents emphasized the desire for mutual commitment and investment in the mentoring relationship, establishment of boundaries early on, and recognition of personhood outside of their role as an academic. Those from historically excluded and minoritized backgrounds shared the importance of a mentor acknowledging the systemic and societal issues that not only impacts the mentee, but the family members with whom mentees may have close connections or responsibilities of caring for. For example, one respondent shared:

…I have family members suffering at the hands of systemic racism (drug/alcohol/gambling addictions, suicide ideation, homelessness, extreme poverty, early death from health disparities etc). And this sadly can be the norm in minoritized communities due to colonialism and systemic racism. I recognize some people in my lab won’t be able to understand, and I feel like I have only been able to connect with other [minoritized] students who may be in similar situations/understand this disparity in the USA. So the drawback is having to be able to do science and keep up while I know my community faces larger issues (sometimes science seems small to me).

Openness: Respondents noted the importance of a mentor listening to learn, asking about the mentee’s interests/needs, soliciting feedback, and maintaining an open, curious outlook. This fosters a process of lifelong learning, critical reflection, and constant adjustment as stated by one participant:

[The relationship] can be enhanced by staying committed to the process, asking for feedback from mentees, and continuing to learn throughout the entire process. No one expects a mentor to be perfect, and mentors from different backgrounds can have a positive impact on mentees, however, it just takes time. It involves speaking up on the inequities in academia and not avoiding the topic as if it doesn’t affect the mentee. And it involves a lot of listening, learning, and reflecting.

Communication: Acknowledgment of differences that may create gaps in communication or understanding was preferred by respondents. These gaps may be more present for mentorship relationships where identity incongruence is a factor. The expectation that the mentee adapt their vernacular to match that of a mentor’s dominant culture or to meet standards of “acceptability” was described as harmful. Respondents also shared the importance of discussing expectations up front. Communication was easiest when there is a safe space, including space for dissenting opinions; this dynamic contributed to comfort and authenticity within the relationship. In reflecting on a positive mentorship experience, one respondent shared:

I was given the freedom to openly express myself and my feelings. Especially in regards to injustices I observe in the neuroscience and academic community. He supported my stance even when the larger majority in the community found me controversial.

Flexibility: Respondents desired a mentor who adapts to their evolving needs over time. A mentor lacking in flexibility was cited as a major barrier to academic development:

My mentorship needs changed as I became more independent, but my advisor didn’t adapt their style with those changes. The mentor was overbearing and wanted me to spend my time on their own projects that weren’t my thesis work. This was very stressful, especially as a person for whom time is limited due to my disability.

##### Theme 2: allyship and management of power imbalance

This theme focuses on the mentor’s status within academia, and society at large, and how the power afforded by the intersections of the mentor’s identities can be used in ways that impact the mentee’s experience in neuroscience.

Understanding and appreciation of lived experiences: Most respondents wanted mentors who seek to understand how multiple, interlocking systems of oppression impact their pursuit of a neuroscience career. One respondent shared the power of their mentor’s support in a time of need:

When you are having financial situations for a second and you can’t seem to get out of it and it is stressing you and impacting your science and personal life…when someone says ‘I can help’ and they do mean it and take the time to understand why and how you got to that situation and offer to help is just amazing—you feel seen and taken care of and that’s what I felt. I felt like I had an ally and someone that would never let me struggle.

Navigation of identity differences/incongruence: For respondents in cross-identity mentorship relationships, they shared that operating from a space of assumptions was detrimental to the relationship, for instance:

[Mentor] doesn’t understand what it’s like being an underrepresented minority in a mainly white space. [Mentor] doesn’t understand that sometimes me not being vocal isn’t a lack of ideas but more just feeling uncomfortable. I am a first-generation college student.

Alternatively, many participants shared that, if navigated appropriately, the cross-identity relationship could serve as a space for new perspectives, collective knowledge, and advocacy.

When speaking on the positives that result from appropriate navigation of differences, one respondent considered how mentorship experiences in the present impact future mentorship and described it as:

[An] opportunity to learn from someone of a different culture/identity. Positive experiences with being a cross-cultural mentee can lead to one being a better cross-cultural mentor in the future.

Mentor’s leveraging of power and positionality: The onus of ensuring the relationship is equitable falls largely on the mentor, whose power can be used to improve conditions within the academy (via influencing institutional culture) or can be used to maintain the status quo or perpetuate harm. Respondents identified several forms of harm within cross-identity mentoring relationships including: gatekeeping, performative allyship, tokenism, and exploitation of labor. One respondent shared about their mentor:

They were also way more advanced in their career and as a result served in a number of leadership roles and had power and influence at the institutional level. As a result of this, they wielded their power in harmful ways and sometimes impacted the upward mobility of me as a mentee via their positionality and status in academia. They were essentially an academic gatekeeper.

Central to a positive relationship is a sense of trust that the mentor will act with the mentee’s best interest in mind. The mentor should be willing to use their power to advance the interests of the mentee and to disrupt oppression within the academy. For example, this may look like intervening when a mentee is asked to perform invisible, uncredited labor that is disproportionately assigned to historically marginalized persons (e.g., community outreach, mentoring other marginalized persons, recruitment of other marginalized persons into the department). Other examples include providing mentees professional opportunities to build skills or academic networks, amplifying their mentee’s work on public platforms and in professional circles, and supporting or speaking up for their mentees in spaces where mentees may not be present (or invited).

Belongingness: Respondents shared the desire to be in community with people who value their existence and contributions. The ability to connect over shared experiences contributed to feelings of validation and being seen in spaces where they are the minority:

My one mentor who is a Black woman in STEM greatly impacts me by providing me with advice on how to navigate academia being a Black woman. She is able to directly relate to problems I face and share her experiences and how she overcame them. She helps me feel validated and seen in ways that I cannot even begin to explain, so she has been very impactful in that realm.

##### Theme 3: academic sponsorship

Academic sponsorship was encapsulated by respondents’ desire for resources to promote their advancement in neuroscience. Respondents wanted their mentor to leverage existing networks to help them build their own network. Respondents additionally desired to learn specific skills that will make them competitive in academia (e.g., grant writing, statistical analysis, publishing manuscripts) but also how to manage critical transitions, such as becoming the Principal Investigator of a research lab. One respondent desired mentorship on:

Transitioning into a mentor/supervisor role: what to look for when hiring Research Assistants (RAs), navigating the transition from mentee to mentor, and becoming the “manager” of a lab group/small scientific team.

They also noted the importance of nomination for awards and introduction to new opportunities as a form of sponsorship.

##### Theme 4: institutional barriers impacting navigation of academia

This theme describes academia as an institution that upholds and recapitulates systems and practices of society at large, particularly those that thrive by the oppression and invisibility of minoritized and diverse beings. Respondents raise the need for specific resources (e.g., securing accommodations for a disability, funding for historically marginalized groups), but often having mentors who are unfamiliar with these options or how to support their mentee in securing them. One respondent recommended mentors:

Get additional training in disability resources for mentees and how mentors can help trainees secure those resources.

Supplemental mentors who are more familiar with how to support their mentee in securing resources may be necessary; however, a number of respondents shared the challenges of having a mentor who is unwilling to acknowledge their shortcomings or unwilling to permit co-mentorship.

#### Mentorship needs by developmental stage

It is critical to emphasize that the needs of a mentee may change as they progress through the academic pipeline. Accordingly, mentorship styles should evolve and adapt as the mentee advances in their educational and professional career and increasingly gain greater independence in their research program. The following outlines mentorship needs identified by survey participants of varying training or early career stages.

##### Graduate students

When seeking mentorship, graduate students rated sponsorship as top priority. Provision of opportunities for financial supports (personal expenses or project-based needs), is particularly important given the limited funds offered by graduate student stipends:

To their credit my mentor helped me secure a prestigious position and continued to nominateme for many awards/professional development opportunities. (I wrote my own recommendation letters for these awards/applications). They also helped me contact student financial services when I was considering dropping out due to family financial struggles and even offered to give me a small [dollar amount] loan to purchase groceries.

The second priority was having a mentor who is willing to consider their perspective and have open conversations about lived experiences. Responses highlighted how establishing mutual understanding did not require congruence across mentor and mentee identities alone, but necessitated appreciation of the complex ways identities intersect to create a power structure that differentially impacts access in academia. One graduate student acknowledged that having some shared aspect of identity with their mentor created an important common ground, but this did not always guarantee understanding:

I felt initially comfortable that she was a queer woman but honestly that was almost a redherring. Just because you share an identity with someone doesn’t mean that they have an intersectional understanding of accessibility and equity within academia.

##### Postdoctoral fellows

For postdoctoral fellows, most important was a mentor’s willingness to consider their perspective and understand their lived experience. One respondent shared the importance of considering whether the mentee is a first-generation student and the financial burden of pursuing an academic career:

Sometimes my mentor assumes I know things about the institution of academia that I don’t because my first exposure to grad school/academia was when I started my PhD. My mentor (along with most of the other faculty) also went to school at a time where student loans weren’t so predatory and overwhelming, so they don’t necessarily understand how big a financial burden it can be.

Mentorship at this juncture may also focus on providing validation and encouragement to mentees with the goal of increasing their sense of self competency to build an independent program of research following fellowship. Given the brevity of many fellowship programs, open communication surrounding satisfaction with mentorship was identified as a tool to strengthen the relationship.

##### Early career

Junior faculty rated sponsorship as most important in what they seek from a mentor. Given early career scientists need to build an academic network and demonstrate high productivity in a relatively short probationary period, their inclusion in pertinent research and professional development opportunities (e.g., grants, collaborations, access to databases) is central to retention. One junior faculty shared:

At my stage of career, I would value the personal connections that my mentor can provide (e.g., connecting me to potential collaborators), informing or sponsoring me in unique opportunities (e.g., co-investigator or collaborator on research projects/grants, editorial opportunities, etc.), and support in grant-writing.

Second in priority was having a mentor who provides coaching of career skills and individualized time and attention. Faculty who did not receive guidance on their science were forced to identify mentorship elsewhere.

#### Additional identity considerations

While the primary focus of analysis was mentorship experiences and needs across career phase, we considered other aspects of participants identities and the intersections of these identities, when appropriate, keeping in mind our limited sample size and a desire to maintain participant anonymity (which influenced the intersectional categories examined). We analyzed the existing themes and subthemes by intersectional categories to highlight some of the salient narratives from the data, but also acknowledge the need for a larger sample to offer a more comprehensive overview of lived experiences. Of particular importance is how a participant’s social locus within society and the academy, based on the intersections of their identities, shapes their experience as neuroscientist mentee. Originating from Black feminist thought (Truth, 1951; Combahee River Collective, 1995), an intersectionality framework extends beyond analysis of a single aspect of a person’s identity (e.g., race, class, gender), acknowledges the multidimensionality of their lived experience, and considers how this experience is influenced by societal power structures (Crenshaw, 1989; Collins, 2000).

Participants in our study living with a disability most frequently identified challenges around navigating identity differences with their mentor. Specifically, they described a culture of ableism that is especially prevalent within STEM fields, which makes advocating for their needs and resources to support their success in neuroscience a challenge and emotionally taxing. Respondents with a disability rarely encountered mentors in neuroscience who also have a disability. They shared that some senior neuroscientists may have non-visible disabilities but opt for nondisclosure due to the stigma and discrimination they may experience. This speaks to how the culture of academia (known to reward high productivity/output, competition, and perfectionism) is not conducive to the psychological safety or vulnerability necessary for this type of personal disclosure. Consequently, this may limit rich opportunities for mentees to connect over shared experiences with a more senior neuroscientist living with a disability. Respondents also shared that cross-identity differences within mentoring relationships can be leveraged in a way that are supportive and introduce new ways of being. For example, one woman living with a disability credited her advisor with helping her to present herself in strongest light possible in an application, countering the social norms and socialization of women to be modest and not self-promote or share accomplishments (Diekman et al., 2010; Smith and Huntoon, 2014).

There was qualitative evidence that racially and ethnically minoritized women in the sample were more likely to discuss an aspect of their identity in relation to their family (e.g., one participant described themselves as a daughter and provider to their mother), to share the challenges of navigating work-family life as an academic, and to disclose some of their personal values about family that may conflict with their mentor’s and the academy at large. For example, academia reinforces values that align with the dominant white male majority and reward academics who adhere to these values and uphold social norms within the academy (Brauer et al., 2022). Greater appreciation is needed of the differences in family structure and caregiving responsibilities, the double duty of motherhood while working as an academic, and the unequal distribution of academic labor—many times invisible—that disproportionately impact racially and ethnically minoritized women (Moore, 2017; Social Sciences Feminist Network Research Interest Group, 2017).

## Discussion

The results of the present study amplify and extend numerous efforts within the neuroscientific community aimed at identifying and dismantling oppressive structures and processes that lead to the systemic exclusion and marginalization of diverse people in neuroscience. We stand in solidarity with organizations, such as (Singleton et al., 2021; Black in Neuro, 2022; SPARK Society, 2022), and the BRAINS program (Brains, 2022) which create visibility for the invisible and/or erased neuroscientists, and uplift perspectives that deviate from the mainstream. Our findings resonate with many other accounts that highlight how race and ethnic identity are reified and reinforced in dynamic ways that feed into the preservation of whiteness, power, and dominance of the majority. Additionally, findings provide rich context and situate the intersectional experiences of neuroscientist mentees from other marginalized and historically excluded backgrounds (e.g., sexually minoritized, disabled, economically disadvantaged, immigrant). Figure 1B provides a conceptual model that illustrates the layered, complex dynamic of a mentor-mentee relationship within academia.

We specifically seek to highlight the individual-level factors that both the mentor and mentee separately bring to the relationship, as well as the clear power differential that is inherent to the interpersonal dynamic. This relationship offers a set of experiences that are nested within a larger ecosystem of the academic institution as well as society and social values (Sambunjak, 2015; Vargas et al., 2021). Both participants of the mentorship dyad bring their own set of worldviews, lived experiences, and biases which influence the discourse and behaviors within the mentorship relationship (DiAngelo, 2018). Interpersonal factors such as degree of individualism vs. collectivism, expectations for the relationship, and social justice orientation (Clutterbuck, 2007; Vargas et al., 2021) vary across culture and other aspects of identity but are important factors that influence the nature of engagement particularly when there is identity incongruence. For mentors, their past experiences as a former mentee, current encounters within the system of academia, willingness to acknowledge their power and privilege, and general openness to identity differences may shape their approach to mentorship (Vargas et al., 2021). For historically marginalized mentees, feelings of imposter syndrome, social isolation, and experiences of discrimination or identity-based stress may impact help-seeking within the relationship (Williams et al., 2018; Muradoglu et al., 2022). Full appreciation of the mentorship dynamic requires careful consideration of individual attributes and experiences within the dyad as well as contextualization within a broader macrosystem of and will likely, at points, require participating in difficult dialogues as a means of engagement in equitable mentoring relations (Madore and Byrd, 2022).

Many of the academic barriers identified by our group are by design in that they recapitulate social hierarchy within the academy and have unfortunately led to a mass exodus of some of the most talented minds (see Flaherty, 2021; Matias et al., 2021 for examples). We acknowledge “the master’s tools will never dismantle the master’s house” (Lorde, 1984) as the foundations of academia are deeply flawed and designed to maintain these inequities. True institutional change will require a multi-tiered approach including implementing change at the policy level. However, engaging in effective mentorship practices offers the opportunity to deconstruct oppressive systems via investing in the mentee’s success, nominating them for leadership roles, creating a sincere environment of inclusion and belongingness, and intentionally changing the demographic landscape of neuroscience even if primarily at the local (laboratory) level. As a mentor, embarking on the ongoing, life-long process of critical self-reflection and education (e.g., developing language to discuss inequities, recognizing one’s role in maintaining and/or perpetuating inequities within academia, naming and calling out epistemic exclusion)–is a crucial first step. Additionally, culturally-responsive mentorship education should be a component of faculty onboarding, and training should be routinely required throughout one’s career in the academy. Mentorship training that is process oriented and moves beyond the theory or strategy of mentorship and delves into personal mentorship experiences, reflections, and application would be especially beneficial (Balmer and Richards, 2012).

The themes in our study underscore the importance of a mentor’s awareness of their status within the larger social hierarchy, acknowledgment of the structural factors that impact diverse mentees (i.e., structural racism and power), and engagement in active allyship, emphasizing elements of academia that extend beyond those that put the onus on diverse mentees. Inaction to address these bare minimums in the face of persisting structural barriers implies complicit endorsement of the processes that cause harm to minoritized people. We offer a list of recommendations for cross-identity mentorship relationships in Figure 1C based on the collective knowledge gleaned from our group survey.

Our qualitative investigation, conducted by diverse neuroscientists about diverse neuroscientists, challenges research epistemology that prioritizes Eurocentric approaches to the production of knowledge, while highlighting the artificial distinction between the “researcher and the researched” (Probst, 2016; Holmes, 2020). We hope this examination of cross-identity mentorship in diverse early-career neuroscientists will inspire future efforts that elevate the diverse voices of those experiencing the effects of structural oppression, as those perspectives hold the transformative and radical insights needed for change.

## Data availability statement

The original contributions presented in the study are included in the article/Supplementary materials, further inquiries can be directed to the corresponding authors.

## Ethics statement

The studies involving human participants were reviewed and approved by University of Minnesota Institutional Review Board. The patients/participants provided their written informed consent to participate in this study. Written informed consent was obtained from the individual(s) for the publication of any potentially identifiable images or data included in this article.

## Author contributions

TH-J wrote the first draft of this manuscript, designed the survey, and completed data analysis. RN designed the survey, completed data analysis, and contributed to writing the methods. CC-I contributed to writing the discussion and created the figure. JA contributed to writing the abstract. All authors contributed to the article and approved the submitted version.

## Abbreviations

BRAINS: Broadening the Representation of Academic Investigators in NeuroScience
CNS: Community of Neuroscientists
GPA: Grade point average
GRE: Graduate Record Exam
NIH: National Institute of Health
NINDS: National Institute of Neurological Disorders and Stroke
PhD: Doctor of Philosophy
STEM: Science, technology, engineering, mathematics
URM: Underrepresented minority
US: United States.
