# Supplementary material for: A developmental approach to diversifying neuroscience through effective mentorship practices: perspectives on cross-identity mentorship and a critical call to action
Source: Front Integr Neurosci. 2023 Feb 8;17:1052418. doi: 10.3389/fnint.2023.1052418 (PMC9944572; doi:10.3389/fnint.2023.1052418)
Supplement: Supplementary file 1 [file Table_1.docx]

| **Subtheme** | **Barriers** | **Facilitators** |
| --- | --- | --- |
| 1. **Approach to Mentorship & Interpersonal Dynamics:** *Interpersonal factors and mentor attributes that facilitate a positive and empowering mentor-mentee relationship.* | | |
| Respect of values and    priorities | **Minimizing trainee’s concerns**  “Sometimes conflict is necessary and downplaying a trainee's legitimate issues is just going to make the trainee not trust that you'll support them.”  **Ignoring differences in work/life balance**  “This mentor was often very critical, unavailable, and often saw my desire for work-life balance and a family life as a reflection of my lack of commitment to my work.” | **Mutuality and shared commitment**  “Also, in order for there to be a good mentorship relationship, both parties need to have mutual respect for each other, especially as it relates to racial/ethnic diversity and cultural differences - this is very important.”  “Both parties have to be willing to compromise (give and take). Boundaries need to be established early on to prevent a toxic/negative mentorship relationship (e.g., not working on the weekends). There should be a list of goals that both parties agree on.”  “Open communication, willingness to take on the other person's perspective, honesty, mutual respect . . . Both parties (mentor and mentee) should also recognize that effort will be required on both ends and that there is a certain level of investment for [the relationship] to be successful.”  **Acknowledge mentee’s personhood outside of academia**  “Their insistence on checking in on me as a person and recognizing my life outside of the lab is another thing that I've loved and wish to do for my own mentees.”  “I have been very fortunate with my mentor. I am generally happy, particularly memorable moments with him is when he will assure me out of nowhere that I am doing a good job. He will provide genuine confirmations. He also assures me that I should take time off to see family/he provides a healthy environment for me to do science.” |
| Openness | **Defensiveness**  “I attempted to communicate feeling less supported, but [my] mentor became somewhat defensive  and cited a bunch of professional development projects they were allowing me to do in addition to my lab work. I didn't do a good job communicating that I needed to feel like my scientific ideas were also valuable and that I felt like my mentor was no longer interested in me running more experiments.” | **Listen to understand**  “Be willing to listen to students that are from a different background than that of your own. Be willing to compromise and understand that you must be flexible as well. Learn to listen more than you speak. Do not cause more undue stress upon your students. Also, even if you think the relationship is copacetic, always check in with your student to see how they view the relationship.”  **Consider mentee’s interests**  “My co-mentor's openness to explore my personal interests in research and trust have been instrumental in making me feel like I belong in science and like my contributions are valuable.” |
| Communication | **Not checking in**  “...this was a contentious relationship, and I did not feel supported by this specific mentor. Also, there was no flexibility or willingness to change. Additionally, this mentor thought they were being helpful, instead, they were causing great harm and never thought to check in with me to see how I felt about our relationship.”  **Overcorrecting language/differences in dialect**  “The mentor consistently tried to correct everything I said or did, as if I could never say or do anything right. I would have to repeat myself several times in different ways, in order for this mentor to understand my perspective and point of view, even when I had explained things clearly the first time; this was very stressful.” | **Acknowledge differences**  “My other mentoring experience could be better if he spoke on my identities.”  “To the mentors that I've had who avoid discussing how my identity shapes my experiences, I implore you to speak up on this topic. A huge part of the mentorship relationship is lost due to this lack of discussion. To those who take initiative to start these conversations, I am very thankful.”  **Create a safe space to speak up**  “Communication and honesty. Being able to freely speak your mind without fear for repercussion is very important. oh and RESPECT - in all senses of the word!”  “Reciprocity and good communication. I was always taught to ‘manage your manager.’  Which is to make sure you and your mentor are always on the same page and being comfortable with vocalizing your immediate needs.”  **Make expectations known (conversations surrounding communication style, feedback, and support needs)**  “She struggled to understand my communication style and need for direct feedback.”  “Maybe more communication on how I can share if I'm stressed, what to do. I don't really know how/where I need support during these times.”  “Clear communication between mentor and mentee on well-being and satisfaction of their relationship.” |
| Flexibility | **Rigidity**  “My mentorship needs changed as I became more independent, but my advisor didn't adapt their style with those changes. The mentor was overbearing and wanted me to spend my time on their own projects that weren't my thesis work. This was very stressful, especially as a person for whom time is limited due to my disability. I would be assured that these projects wouldn't take much time, but then I'd be assigned much more time than we had agreed to on these peripheral projects and they took precedence over my own work. These experiences taught me a lot about my own boundaries and how to navigate difficult situations (I'm working on it).” | **Remain adaptable**  “The responsibilities of the mentor should change and grow with the needs of the mentees”  “My advisor is flexible about working from home and providing accommodations.” |
| 1. **Allyship and Management of Power Imbalance:** *Mentor’s recognition and leveraging of their privilege, power and advantages to support the mentee’s professional and personal developmental; the desire to learn about and show appreciation for mentee’s lived experiences despite incongruences across identities.* | | |
| Understanding and    appreciation of lived    experiences | **Perpetuation of “isms” (e.g., ableism, racism, sexism)**  “There are very few disabled individuals in STEM, and STEM fields in general have a strong culture of ableism. Although I generally like my primarily mentor and have a good relationship with them, we can be on entirely different wavelengths when it comes to reasonable expectations for productivity when I am dealing with a major health issue. I often feel guilty that I am letting my mentor down when my productivity is impaired due to illness. This guilt is often self-imposed, but additional support from mentors can help mitigate this.” | **Use privilege and power to support**  “I was having a very shitty day (as per usual) about my science but also moving at the same time and needing to do a presentation in a couple of weeks and this person offered to 1) help me with my payments and 2) listen to my presentation over and over. And this may not sound as much, but when you are having financial situations for a second and you can't seem to get out of it and it is stressing you and impacting your science and personal life….when someone says ‘I can help’ and they do mean it and take the time to understand why and how you got to that situation and offer to help is just amazing - you feel seen and taken care of and that's what I felt. I felt like I had an ally and someone that would never let me struggle.” |
| Navigation of Identity Differences/Incongruences | **Assumptions**  “On top of the fact that he has been writing NIH grants for [number] years, he grew up in an area where many people had graduate degrees and went to very high level schools for undergrad/grad school/postdoc. Due to this, he is intimately familiar with how academia works and kind of operates on the assumption that others are too.”  **Requiring additional labor/asking mentee to educate**  “Educating them and bringing awareness to topics they should already be aware of or sensitive about. The form of communication can also be a challenge.”  **Not seeking understanding**  “Doesn't understand what it's like being an underrepresented minority in a mainly white space. Doesn't understand that sometimes me not being vocal isn't a lack of ideas but more just feeling uncomfortable. I am a first-generation college student.” | **New perspectives**  “Different perspectives. But also novel approaches to dealing with different situations. it is also a different networking experience and circle of people that can be beneficial.”  **Advocate for a strengths-based presentation of self**  “Mentor helped me present myself in the strongest possible light. Women are often trained to be modest and not brag about our accomplishments. White, male mentor helped me construct a more confident, assured narrative for my accomplishments (ex: draft a more impressive cover letter).”  **Contribute to collective knowledge**  “Diversity in all forms is important.  Having a mentor, like having a friend with a different identity, results in growth and understanding.  Sharing different experiences and perspectives strengthens collective knowledge.”  **Reinforce positive mentorship practices**  “Opportunity to learn from someone of a different culture/identity. Positive experiences with being a cross-cultural mentee can lead to one being a better cross-cultural mentor in the future. If [the] mentor is from a privileged identity, they can use their position to advocate for mentees with less privilege.” |
| Mentor’s leveraging of power and positionality | **Performative allyship**  “Me and the mentor I have through my school have a very positive relationship for the most part. However, we began to clash when his mentoring style changed, becoming toxic, degrading, and detrimental to my mental health as well as that of other students in the lab. Our relationship became very rocky when they told me that a grad student who I am very inspired by only got a post-grad job because they were Black. Saying things like this are extremely harmful. To make it worse, this mentor stood as a faculty liaison for our school's student-run diversity group. It made it very clear to me that a lot of faculty members may be participating in these groups for reasons that are performative and superficial rather than because of their passion for diversity.”  **Use of harmful language**  “The deficits to my mentor experience are that sometimes, the language that is used to speak on historically marginalized populations is harmful. It gives me anxiety and gets me very angry, to a point where I no longer wish to be around that person or in school in general. Too many people refuse to realize their biases and reflect and learn how to truly be anti-racist. This lack of realization can be so, so detrimental to students.”  “Microaggressions are a real thing and it is not okay to write it off as a bad day.”  **Tokenism**  “Wondering whether you have been tokenized. Questioning the mentor's motives for taking you on as a ‘diverse’ person.”  **Exploitation of labor/power imbalance**  “Pressuring trainee to work unreasonable hours, often without pay…Continually dismissing trainee's work and leaving them off papers.”  “Power imbalance used as a weapon, not being able to meet with mentor, difference in expectations for one another, not feeling able to discuss needs, being culturally incompetent or racist or sexist etc.” | **Open doors for mentee**  “Another benefit, especially if your mentor is a white man, is that, in our society, many people think highly of their opinions and recommendations, meaning that they can open many doors to their respective mentees. Having a white man advocate for you has the potential to expand your network in a big way.”  “Feeling like they are saying my name in a positive way in rooms I wasn't in.”  **Foster trust**  “There are also strengths in identity incongruence, but only if this mentorship relationship is handled properly. One strength I mentioned before is that it has the potential to open many doors and connections for the mentee. And, if the mentors really take the time to listen, learn, and understand how their mentee's identity may affect their access to certain resources or opportunities, it can build a strong sense of trust and appreciation within this relationship.”  “Maybe most importantly, the mentor needs to be able to trust the mentee to do what's best for them and understand that the mentee might not always take/need/want their advice. The best mentors learn from mentoring others.”  “Trusting and honoring relationship. Constant availability for support. He hypes me up and my science. He understands my project really well, and is open to discussions all the time. He encourages me to try new experiments or to apply to grants etc. He has been helpful in writing grants etc.”  **Equitable relations**  “To date, mentors rarely allow full autonomy in my decision-making process. This probably has a lot to do with age and patriarchy. This is one of the reasons why I ultimately changed primary advisors (I felt as if I didn't have a voice and was left out of key decision-making conversations). Because this current mentor (not advisor/PI) shares race/ethnic identities with me, I believe it is more equitable when we meet and discuss my experiences.”  **Protection from harm**  “I need a mentor that is unafraid of standing up for me when I am not around and need to be protected.” |
| Belongingness | **Lacking diversity in lab and academic institution**  “We do not have a Hispanic/Black faculty in our department. That’s the biggest barrier - not being able to even reach out to someone because there is nobody to reach out to.”  “There are many barriers we confront including a dearth of Native and Indigenous scientists in western academia. I know of one Native neuroscientist Principal Investigator and they aren't at my institution. There is also a disparity of SWANA (Southwest Asian and North African) women in neuroscience. There exists the broader issue of SWANAs not being recognized as minoritized individuals in academia which further contributes to the assumption that they exist and have the same experiences as their white male counterparts. This thinking that is pervasive toward SWANA and AAs (Asian Americans) contributes to feelings of not belonging.”  “The major drawbacks include the fact that they cannot always relate to your experiences, and because of that, the way your identity shapes those experiences can often be overlooked. Also, someone whose identity differs from yours might not be able to provide the best advice on how to navigate a certain problem. Further, they might not even understand just how deep-rooted institutional racism is and may not think problems you voice are a big deal. It would take real commitment and a lot of learning on their part to be a mentor who can listen and provide insight and resources, despite not sharing your personal identities.”  “There are very few openly disabled individuals at the tenure-track level in STEM. Those who are disabled often became disabled late in life, resulting in a very different experience compared to those who were disabled from birth/childhood. Even within the disability community, there is extensive internalized ableism and a sense of competition for ‘who is the most disabled.’ This results in ignoring access needs and creating unnecessary barriers for disabled trainees. I didn't meet an openly disabled STEM professor until graduate school.” | **Connecting on shared or similar lived experiences**  “Being at relatively similar career stages (postdoc mentor, PhD student mentee) made it easier to relate to one another and support one another in the lab.”  “Sometimes mentors are formally assigned and this is not always the best match as I have found that having a shared aspect of identity is often a good entry point into a relationship or developing some sort of understanding.”  “The strengths of mentors who share the same identity and background as you are that you can directly relate to them, and they serve as a tangible representation that you too can be where they are in life. They help show you that you can get to your ideal job even with the various barriers you may face. They show that it is possible.”  **Encourage and embrace authenticity**  “Feeling like you have to maintain ‘double consciousness’ where you are thinking about what they are thinking … Sometimes unintentional feelings like you have to make yourself more watered down or palatable … Feeling like you may not fit in especially if the mentor's identity is similar to the majority.” |
| 1. **Academic Sponsorship and Provision of Resources:** *Offering mentees opportunities to build skills and collaborative networks necessary for independent academic work and advocating for them in spaces where they are not commonly invited.* | | |
|  | **Lack of time/individualized attention**  “I believe that some people should only mentor 1 to 2 students. They get overwhelmed by more than that which decreases their relationship and attention towards other mentees. Mine is definitely not equipped to handle more than 2-3 mentees.” | **Coach practical skills**  “Anecdotal and data-driven solutions for life/career challenges. Advice on next steps and best practices for navigating grant writing, publishing, and attending national and international conferences, and collaborations (how to balance these important aspects of career advancement).”  “She offered me many opportunities to be on papers, supported me by connecting me with subsequent mentors and served as a referee for work/graduate school, and offered me opportunities to hone my experimental skill sets in Event Related Potential (ERP) and imaging data collection.”  “She had high expectations of me and helped me give good talks. I learned a lot on how to present science. She provided good lab/experimental goals.”  **Networking**  “Particularly in the past year, he has been incredibly helpful and invested in helping me advance my career in multiple aspects, but especially networking. Through his connections, I was informed of a funding opportunity through a foundation that funds rare disease research, with matching funds from [association]. I received the award which has put me in contact with other scientists involved in the [association]. As a result, I have been invited to attend the Advisory Board meeting prior to the upcoming [omitted] meeting where I will meet many other prominent scientists.” |
| 1. **Institutional Barriers Impacting Navigation of Academia:** *Guidance in bridging voids in mentorship  and accessing resources (funds, laboratory space, technical assistance, etc.) to support mentee’s training and academic advancement.* | | |
|  | **University-level barriers**  “Both mentors are at R1 institutions and cannot relate to the struggles I face working at a non-R1 institution in terms of lack of resources and support.”  “…there are few Black women who exist in this space and sometimes none that exist in a given department. Additionally, for this very reason, it is common that a lot of other people will want them to serve in a mentorship role. There is also the reality I must acknowledge that people of color in academia are also taxed with these informal mentorship roles which becomes an invisible burden and can also hinder the mentor's professional advancement (so being mindful of this as a mentee is important and ‘not wanting to bother them too much’ is an additional layer to navigate)...Figuring out how to maintain multiple relationships and getting everything I need from mentors (formally and informally) assigned can be a challenge.” | **Understand resources specific to mentee’s needs; understand institutional infrastructure**  “Get additional training in disability resources for mentees and how mentors can help trainees secure those resources.”  “Better access to resources that may be specific to URMs- training, funding, maybe helping me to connect with faculty mentors so that there might be less differences.”  **Acknowledge supplemental mentors may be necessary and help identifying them**  “No support in finding another mentor. My mentor's leadership position was also a conflict of interest that made it hard for me to find another mentor as others may perceive taking this role might slight them.”  “I went to a small college where neuroscience wasn't a major so I don't have much connections in the field from college…There are very few people (faculty researchers) that I can make those connections with. Also when their scientific interests do not overlap it's hard to make those connections.”  “The mentor was a barrier because she refused to let me be mentored by anyone else.” |
